# Effect of Back-Gate Voltage on the High-Frequency Performance of Dual-Gate MoS_2_ Transistors

**DOI:** 10.3390/nano11061594

**Published:** 2021-06-17

**Authors:** Qingguo Gao, Chongfu Zhang, Ping Liu, Yunfeng Hu, Kaiqiang Yang, Zichuan Yi, Liming Liu, Xinjian Pan, Zhi Zhang, Jianjun Yang, Feng Chi

**Affiliations:** 1 School of Electronic Information, University of Electronic Science and Technology of China Zhongshan Institute, Zhongshan 528402, China; gqgemw@163.com (Q.G.); liuping49@126.com (P.L.); shanhuyf@163.com (Y.H.); 201811022515@std.uestc.edu.cn (K.Y.); yizichuan@zsc.edu.cn (Z.Y.); limingliu@uestc.edu.cn (L.L.); xinjpan@163.com (X.P.); zz001@zsc.edu.cn (Z.Z.); sdyman@uestc.edu.cn (J.Y.); chifeng@semi.ac.cn (F.C.); 2 School of Information and Communication Engineering, University of Electronic Science and Technology of China, Chengdu 611731, China

**Keywords:** MoS_2_, radio-frequency transistors, contact resistance, dual-gate

## Abstract

As an atomically thin semiconductor, 2D molybdenum disulfide (MoS_2_) has demonstrated great potential in realizing next-generation logic circuits, radio-frequency (RF) devices and flexible electronics. Although various methods have been performed to improve the high-frequency characteristics of MoS_2_ RF transistors, the impact of the back-gate bias on dual-gate MoS_2_ RF transistors is still unexplored. In this work, we study the effect of back-gate control on the static and RF performance metrics of MoS_2_ high-frequency transistors. By using high-quality chemical vapor deposited bilayer MoS_2_ as channel material, high-performance top-gate transistors with on/off ratio of 10^7^ and on-current up to 179 μA/μm at room temperature were realized. With the back-gate modulation, the source and drain contact resistances decrease to 1.99 kΩ∙μm at *V*_bg_ = 3 V, and the corresponding on-current increases to 278 μA/μm. Furthermore, both cut-off frequency and maximum oscillation frequency improves as the back-gate voltage increases to 3 V. In addition, a maximum intrinsic *f*_max_ of 29.7 GHz was achieved, which is as high as 2.1 times the *f*_max_ without the back-gate bias. This work provides significant insights into the influence of back-gate voltage on MoS_2_ RF transistors and presents the potential of dual-gate MoS_2_ RF transistors for future high-frequency applications.

## 1. Introduction

Since the first exfoliation of atomically thin graphene [1], two dimensional (2D) materials have demonstrated a wide range of remarkable properties for applications in future ubiquitous electronics [2,3]. Compared to bulk materials, their atomic-scale thickness provides a greater degree of electrostatic control, demonstrating the possibility of ultra-short channel devices with low power consumption [4]. As the most widely studied 2D material, graphene has shown great potential for device applications including high-frequency electronics, flexible electronics, spintronics, nanoelectromechanical systems, and energy storage due to its unique physical properties [5,6,7,8,9,10,11,12]. However, graphene does not have a band gap to limit its application in digital logic devices, and it also limits the maximum oscillation frequency of graphene radio-frequency (RF) transistors. Although band gap can be opened in graphene by artificial nanostructuring, chemical functionalization, etc., those processes add extra complexities with respect to practical applications [13]. Alternatively, another class of 2D material, called transition metal dichalcogenides (TMDCs) (MoS_2_, WS_2_, MoSe_2_, and WSe_2_), not only exhibits many graphene-like properties, such as mechanical flexibility, electrical properties, chemical stability, and the absence of dangling bonds, but also possesses a substantial band gap. TMDCs benefit from a rich pool of elements, and thus they can significantly adjust their electrical properties from metal to semiconductor by forming different compounds. A distinct feature of TMDC semiconductors is that the corresponding energy band structure changes from an indirect band gap to a direct band gap when the material thickness decreases from bulk material to monolayer. They show a wide range of bandgap modulation capability because of rich choices of chemical components, which enables the electronic application of various kinds. As the most studied TMDC material, MoS_2_ has a non-zero band gap structure similar to bulk silicon, making it an ideal choice for making next-generation electronic and optoelectronic applications [4,14,15,16,17,18,19,20]. 

With technological advancements, the high-frequency performance of MoS_2_ devices has attracted tremendous attention [16,18,21,22,23,24]. The high-frequency performance of MoS_2_ RF transistors has been improved through optimizing structure such as self-aligned gate, embedded gate and edge-contacted, etc. [24,25,26]. In 2014, exfoliated MoS_2_ RF transistors with self-aligned gate demonstrated intrinsic cut-off frequency *f*_T_ of 42 GHz and maximum oscillation frequency *f*_max_ of 50 GHz were reported [25]. In 2015, Krasnozhon et al. introduced edge-contacted in exfoliated trilayer MoS_2_ RF transistors, obtaining a high extrinsic *f*_T_ of 6 GHz and intrinsic *f*_T_ of 25 GHz [26]. In 2017, with an optimized embedded gate structure, chemical vapor deposition (CVD) monolayer MoS_2_ transistors with extrinsic *f*_T_ of 3.3 GHz and *f*_max_ of 9.8 GHz were fabricated [24]. In 2018, based on high-quality CVD bilayer MoS_2_, high-frequency MoS_2_ transistors with extrinsic maximum oscillation frequency of 23 GHz were demonstrated [16]. Gigahertz frequency mixer and amplifier based on MoS_2_ high-frequency transistors were also constructed for potential RF circuit applications [16,27]. Those works demonstrated the potential of 2D MoS_2_ for future novel high-frequency electronics. Although the high-frequency performance of MoS_2_ RF transistors has made exciting advances, its cutoff frequency and maximum oscillation frequency are still lower than those of modern Si transistors, and the high-frequency performance of dual-gate MoS_2_ transistors has not yet been reported. 

In this dual-gate structure, the source and drain contact resistances can be modulated via the back-gate voltage, and the influence of the contact resistance on the direct-current (DC) and high-frequency performance of the device can be clearly resolved [28]. Bolshakov et al. presented a near-ideal subthreshold swing of ~60 mV/dec and a high field effect mobility of 100 cm^2^/Vs based on dual-gate MoS_2_ transistors with sub−10 nm top-gate dielectrics [29]. Lee et al. modulated the contact resistance and threshold voltage of dual-gate MoS_2_ transistors with h-BN as gate dielectric through back-gate electrostatic doping [30]. Li et al. demonstrated a high photoresponsivity of 2.04 × 10^5^ AW^−1^ with dual-gate MoS_2_ phototransistors [31]. The dual-gate structure could also be used to investigate the effect of different dielectric interface on the device performance [32]. In addition, based on the dual-gate structure, graphene RF transistors with improved high-frequency performance by reducing the contact resistance using electrostatic doping have been demonstrated [33,34]. Thus, the influence of back-gate voltage on the high-frequency performance of MoS_2_ RF transistors needs further investigation, which is of great significance for further improving the RF performance of MoS_2_ transistors. 

In this study, we fabricated dual-gate MoS_2_ RF transistors with a top-gate length of 190 nm based on the CVD grown bilayer MoS_2_. The static and high-frequency characteristics of dual-gate devices were systematically investigated. The contact resistances of the fabricated dual-gate devices under different back-gate voltages were extracted. A clear modulation of contact resistance *R*_c_ under the electrostatic doping of back-gate was demonstrated. Both DC and RF performance were improved under the electrostatic doping of back-gate. The electrical measurement of our dual-gate high-frequency MoS_2_ transistors at *V*_bg_ = 3 V demonstrated a large current density of 278 μA/μm, a high intrinsic cut-off frequency of 19 GHz and maximum oscillation frequency of 29.7 GHz.

## 2. Materials and Methods

**Figure 1:**
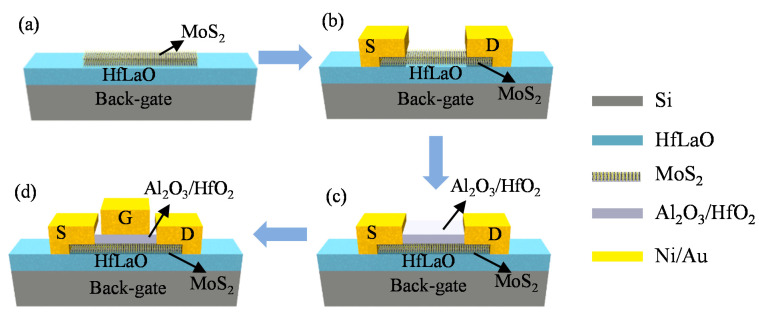
Process for fabricating the dual-gate MoS_2_ field-effect transistors. (**a**) Bilayer MoS_2_ is first transferred on HfLaO/Si substrates. (**b**) Source and drain contact metal deposition. (**c**) Top-gate dielectrics of Al_2_O_3_/HfO_2_ deposition. (**d**) Top-gate metal pattern and deposition. S: source, D: drain, G: gate.

**Figure 2:**
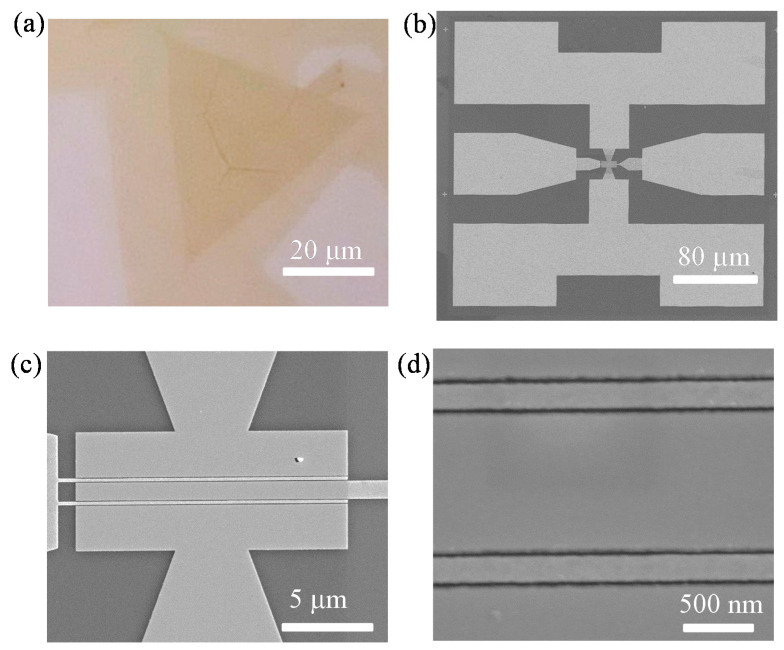
(**a**) The transferred bilayer MoS_2_ on HfLaO/Si substrates. (**b**–**d**) SEM images of the 190 nm MoS_2_ RF transistor with two-fingers structure showing excellent alignment.

Chemical-vapor-deposited bilayer MoS_2_ was used as the channel material in the dual-gate MoS_2_ RF transistors as it has higher carrier mobility, lower contact resistance and improved low-frequency noise when compared with CVD monolayer MoS_2_ [16,27,35]. Additionally, the CVD method is one of the most promising methods for synthesizing large areas and high-quality MoS_2_. The CVD bilayer MoS_2_ films were grown on soda-lime-silica glass substrates with 1.4 g sulfur and 1.5 mg MoO_3_ as the precursors at atmospheric pressure. The details about the CVD bilayer growth process, material imaging and crystal structure characterization have been reported in our previous works [16,27]. After the CVD growth process, bilayer MoS_2_ films were transferred onto highly resistive Si substrates with atomic-layer-deposited (ALD) 20-nm HfLaO. Here, high-resistance Si was used as the back-gate electrode and ALD HfLaO as the back-gate dielectric. As reported in previous work [16,36,37], HfLaO with high dielectric constant could provide improved interface quality and better electrostatic control with the MoS_2_ channel, which is helpful for improving the DC and RF performance of the MoS_2_ transistors. Figure 1 illustrates the fabrication process of dual-gate MoS_2_ transistors. The fabrication of the MoS_2_ devices typically starts after the MoS_2_ films are transferred on top of the HfLaO/Si substrates. Figure 2a presents the MoS_2_ films on HfLaO/Si substrates after being transferred. Then, as shown in Figure 1b, 20/60 nm Ni/Au metal stacks were deposited by electron beam evaporation (EBE) as the source and drain contact electrodes of MoS_2_ dual-gate transistors. In this process, the samples were loaded into the E-beam evaporator (ALPHA-PLUSCO.Ltd., Ebeam-500S Pohang, Korea), and it was waited until the system reaches the pressure lower than 9 × 10^−6^ torr to start the deposition. The deposition rate of 20 nm Ni and 60 nm Au was used as 1 Ǻ/s for both materials. The electrical isolation between different transistors was achieved by performing O_2_ plasma etching for 30 s under an RF power of 50 W with a mixed gas flow of 20 sccm O_2_ and 80 sccm Ar.

The top gate dielectric of the transistors is an important medium for static control of the channel through the top gate electrode, and it has a very important influence on the static and high-frequency performance of the device. The top-gate dielectric is similar to the substrate dielectric, which will scatter the MoS_2_ channel carriers, and the dielectric defects will also capture and release channel electrons. Because of the lack of dangling bonds on the surface of 2D materials, growing high-quality dielectrics on top of MoS_2_ has always been a challenging process [38,39], due to the adsorption of the ALD precursors on a 2D MoS_2_ surface often being more difficult than on conventional semiconductors with a 3D lattice, where plenty of dangling bonds are able help the adsorption during the ALD process. In this work, a two-step seed and growth processes were used in the formation of high-*k* top-gate dielectrics. First, a 2-nm Al layer was deposited on the MoS_2_ surface by EBE and then naturally oxidized in the air to form a 6-nm Al_2_O_3_ layer. Then, 11 nm of HfO_2_ was deposited by ALD using O_3_ as the O source and tetrakis-ethylmethylaminohafnium (TEMAHf) as the Hf source. Finally, the top-gate metal was formed with 20 nm Ni/60 nm Au metal stack by EBE. In the above fabrication process, the patterns of the source, drain and gate electrodes were written using electron beam lithography. In this process, poly(methylmethacrylate) (PMMA) 950 A4 was spin-coated on the substrates at 3000 rpm for 60 s and baked at 180 °C for 180 s. The electron beam was set to a 3 nA current with an exposure dose of 800 µC/cm^2^. Then, the pattern was developed in a 3:1 ratio of isopropyl alcohol (IPA) to methyl isobutyl ketone (MIBK) for 50 s, rinsed with IPA for 60 s, and dried with nitrogen gas. After the EBE deposition of electrodes, lift-off was performed in a beaker of acetone heated to 50 °C for 30 min. Then, the sample was rinsed with IPA and dried with a nitrogen flow. Figure 2b–d display the top scanning electron microscope (SEM) views of the dual-gate MoS_2_ RF transistors with 190 nm top-gate length. The width of the two-fingers top-gate is 30 µm.

## 3. Results and Discussion

### 3.1. DC Characterization

**Figure 3:**
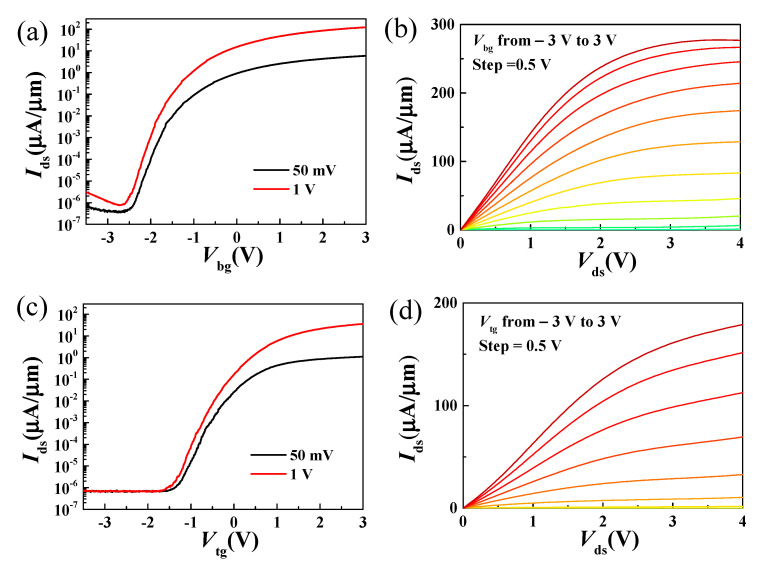
(**a**,**b**) Transfer and output characteristics of the MoS_2_ dual-gate transistors from the back-gate controls. (**c**,**d**) Transfer and output characteristics of the MoS_2_ dual-gate transistors from the top-gate controls.

Figure 3a,c show the transfer characteristics of the dual-gate MoS_2_ transistor from both the back and top-gate configuration. High on/off ratios greater than 10^7^ were achieved for both the back and top-gate modulation. Compared to graphene transistors, this superior on/off ratio is due to the larger band gap [40]. Figure 3b,d show the output characteristics under varied back and top-gate voltages. The gate voltages were varied from −3 V to 3 V with a 0.5 V step. Maximum on-current densities were observed at *V*_ds_ = 4 V are 277 µA/µm and 179 µA/µm for back-gate and top-gate modulation, respectively. The achieved maximum on-current density from back-gate is about 1.6 times the magnitude of that from the top-gate. This comes from the different configuration of back-gate and top-gate devices. As shown in Figure 1d, it can be seen that the highly resistive Si substrate has global control over the entire bilayer MoS_2_ film. Since the channel carriers in the bilayer MoS_2_ films accumulate with increasing back-gate voltage, it can be assumed that the bilayer MoS_2_ is electrically doped under the effect of the back-gate voltage, which further leads to a reduction in the contact resistance between the source/drain (Ni/Au) and the bilayer MoS_2_ film. In the case of top-gate configuration, the gate can only modulate the MoS_2_ films underneath the gate metal [28,33,34]. In addition to the different gate structures, the different top and bottom dielectric layer may also play a critical role in determining the difference of DC measurement [19,32,41] and which need further investigation. In addition, a field-effect mobility of 15.8 cm^2^/Vs was obtained from back-gate measurement by using the relation $\mu_{FE}=\frac{g_{m}L}{WC_{ox}V_{ds}}$, where the back-gate capacitance *C_ox_* is 0.8 µF/cm^2^.

**Figure 4:**
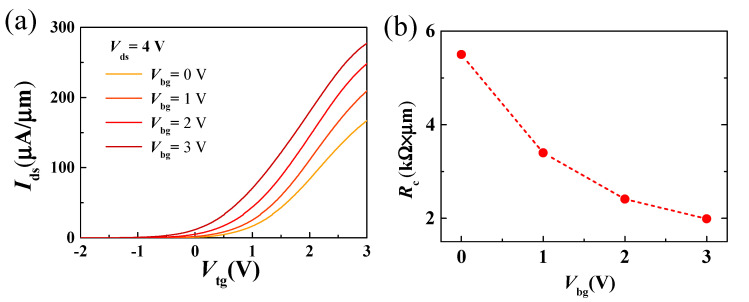
(**a**) Transfer properties of the dual-gate MoS_2_ transistors obtained by sweeping the top-gate voltage with varying back-gate biases at *V*_ds_ = 4 V. (**b**) Extracted contact resistance as a function of back-gate voltage.

Figure 4a shows the transfer curves of a dual-gate MoS_2_ transistor with sweeping top-gate voltage at varied back-gate voltages. With the back-gate voltage increasing from 0 V to 3 V, the on-current density increases from 166 to 278 µA/μm, and the threshold voltage *V*_th_ negatively shifts from 1.1 to 0.1 V. To estimate contact resistances of dual-gate MoS_2_ transistors under different back-gate voltages, an interpolation method reported in previous work was adopted [35,42]. In this interpolation method, contact resistances at different *V*_bg_ were extracted by extrapolating the drain-to-source resistance vs. 1/(*V*_tg_ − *V*_th_), which contains the contribution from metal/MoS_2_ contact and the regions between top-gate and source/drain electrodes. The dependence of the contact resistances versus *V*_bg_ is shown in Figure 4b. The extracted contact resistance is 5.5 kΩ∙μm at *V*_bg_ = 0 V, and decreases to 1.99 kΩ∙μm at *V*_bg_ = 3 V. The reduced *R*_c_ and increased on-current at larger *V*_bg_ can be attributed to the increased electrostatic doping carriers of bilayer MoS_2_ in both the MoS_2_/metal contact region and channel region [28,31,33,34].

### 3.2. RF Characterization

The high-frequency performance of dual-gate MoS_2_ transistors can be evaluated by the cutoff frequency (*f*_T_) and the maximum frequency of oscillation (*f*_max_), which can be obtained from the measured S-parameters [43,44]. The cutoff frequency is where the short-circuit current gain |*h*_21_| equals unity. The short-circuit current gain |*h*_21_| can be defined as:

(1) $$h_{21}=\frac{-2S_{21}}{(1-S_{11})(1+S_{22})+S_{12}S_{21}}.$$

**Figure 5:**
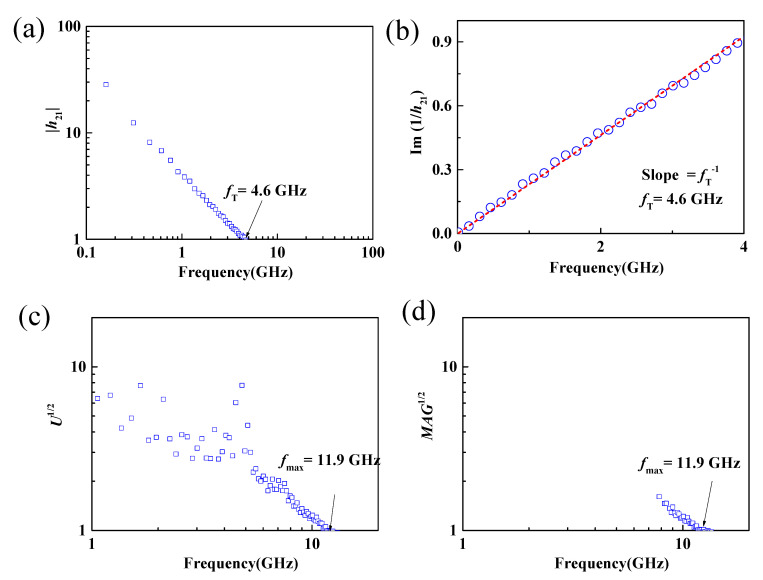
(**a**,**b**) Small-signal current gain |*h*_21_| and Im(1/*h*_21_) versus frequency. Extrinsic *f*_T_ of 4.6 GHz can be extracted. (**c**,**d**) The corresponding unilateral power gain and maximum available power gain versus frequency. An extrinsic *f*_max_ of 11.9 GHz can be extracted.

Similarly, the maximum frequency of oscillation was found when the unilateral power gain *U* was unity, where the *U* can be defined as:

(2) U=|S21S12−1|22K|S21S12|−2Re(S21S12),

where *K* is the stability factor and $K=\frac{1+|S_{11}\times S_{22}-S_{12}\times S_{21}{|}^{2}-|S_{11}{|}^{2}-|S_{22}{|}^{2}}{2\times |S_{12}\times S_{21}|}$. On-chip microwave measurements from 100 MHz to 30 GHz of the dual-gate MoS_2_ RF transistors were carried out using vector network analyzers (N5225A, Agilent (Keysight), Colorado Springs, CA, USA). Before the S-parameter measurement, the on-chip measurement system was calibrated according to the short-open-load-through (SOLT) method using standard impedance calibration samples. Then S parameters of the MoS_2_ transistors were measured, and the short-circuit current gain and the unilateral power gain can be calculated by Equations (1) and (2). As shown in Figure 5a,c, the *f*_T_ and *f*_max_ of the 190 nm MoS_2_ RF transistors with back-gate floating were 4.6 and 11.9 GHz, respectively. The achieved *f*_T_ of 4.6 GHz and *f*_max_ of 11.9 GHz were also further verified using Gummel’s method [45] and maximum available power gain (MAG) [46], as shown in Figure 5b,d. The obtained cut-off frequency and maximum oscillation frequency were consistent with our previous reported work [16], demonstrating the potential of CVD bilayer MoS_2_ for large-scale high-frequency circuit applications [27,35].

**Figure 6:**
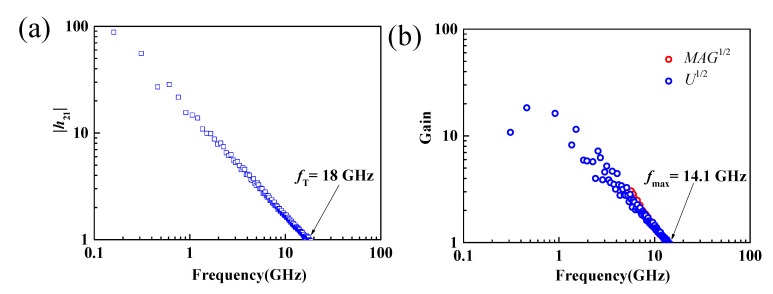
(**a**) Small-signal current gain |*h*_21_| versus frequency, (**b**) unilateral power gain and maximum available power gain versus frequency. Intrinsic *f*_T_ and *f*_max_ of 18 and 14.1 GHz could be extracted.

Although the implementation of the standard calibration method can move the measurement reference plane from the internal receiver of the vector network analyzer to the tip of the ground–signal–ground (GSG) probe, the parasitic capacitance, inductance, and resistance of the test electrodes also have a significant effect on the obtained S-parameters [27,47]. To eliminate the influence of the test electrodes on the measured S-parameters and to obtain the intrinsic RF performance of the MoS_2_ RF transistor, this work uses the standard “open” and “short” structures for de-embedding [25]. Then, the measured S-parameters were converted to Y-parameters, and the de-embedding process was performed under the following equation: $Y_{\mathrm{int}}={\left[{\left(Y_{DUT}-Y_{open}\right)}^{-1}-{\left(Y_{short}-Y_{open}\right)}^{-1}\right]}^{-1}$, where *Y_DUT_* stands for the Y-parameter of the measured transistors. The short-circuit current gain, unilateral power gain, and maximum available power gain versus frequency after de-embedding of the MoS_2_ transistors with gate length of 190 nm are shown in Figure 6. Intrinsic *f*_T_ and *f*_max_ of 18 and 14.1 GHz were achieved, respectively.

**Figure 7:**
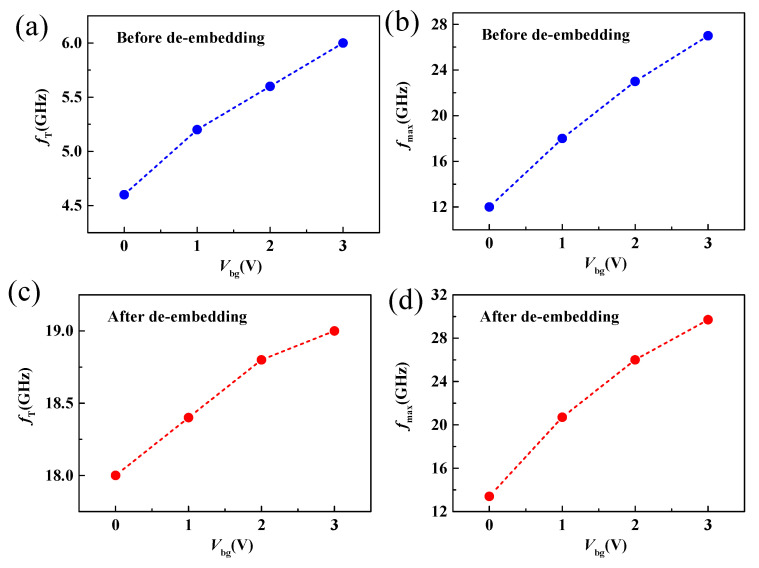
(**a**,**b**) Extrinsic *f*_T_ and *f*_max_ as a function of *V*_bg_. (**c**,**d**) Intrinsic *f*_T_ and *f*_max_ as a function of *V*_bg_.

To improve the high-frequency performance of MoS_2_ RF transistors, we can derive the dependence of *f*_T_ and *f*_max_ on the physical parameters of the device through the small-signal equivalent circuit model, and write them as Equations (3) and (4).

(3) $$f_{T}=\frac{g_{m}}{2\pi }*\frac{1}{\left(C_{gs}+C_{gd})[1+g_{ds}(R_{s}+R_{d})]+C_{gd}g_{m}(R_{s}+R_{d}\right)}$$

(4) $$f_{\max}=\frac{f_{T}}{2\sqrt{g_{ds}(R_{s}+R_{d})+2\pi f_{T}C_{g}R_{g}}}$$

where *g_m_* is the transconductance and represents the channel current controlling capability of the gate voltage, *g_ds_* is the output conductance, *C_gs_* and *C_gd_* is the gate-to-source and gate-to-drain capacitance, respectively. *R_s_*, *R_d_* and *R_g_* are the source, drain, and gate resistances. From Equations (3) and (4), we can see that *g_m_*, *g_ds_*, *R_s_* and *R_d_* play an important role in the high-frequency performance of RF transistors. Therefore, back-gate modulation could be an effective approach for improving the high-frequency performance of MoS_2_ RF transistors. Figure 7 shows the intrinsic and extrinsic cut-off frequency and maximum oscillation frequency of the device as a function of the back-gate voltage. As shown in Figure 7a,c, when the back-gate voltage changes from 0 V to 3 V, the extrinsic and intrinsic cut-off frequencies before and after de-embedding increase from 4.6 to 6 GHz and from 18 to 19 GHz, respectively, demonstrating an obtained peak *f_T_* increase as the increase of back-gate voltage. The improvement of *f_T_* can be attributed to the reduced contact resistance thus improve transconductance and on-current with increasing *V_bg_*, as shown in Figure 4. From the intrinsic *f_T_* of 19 GHz at *V_bg_* =3 V, a saturation velocity of 2.3 × 10^6^ cm/s is obtained, which is comparable with previously reported works [16,25]. Similarly, when the back-gate increases from 0 to 3 V, the extrinsic and intrinsic maximum oscillation frequencies before and after de-embedding increase from 12 to 27 GHz and from 13.4 to 29.7 GHz, respectively. Because the dependence of *f*_max_ on output conductance is more sensitive, the increase of *f*_max_ with increasing *V_bg_* is larger than *f_T_* [34]. Furthermore, a comparison between reported MoS_2_ RF transistors with comparable gate length [22,23,24] is listed in Table 1, below, demonstrating the advantage of dual-gate MoS_2_ RF transistors.

## 4. Conclusions

In summary, for the first time, a systematic investigation of a dual-gate MoS_2_ RF transistor based on CVD bilayer MoS_2_ was performed. Improved on-current and contact resistance performance by optimizing the back-gate voltage were demonstrated. A high on-current of 278 μA/μm and a low contact resistance of 1.99 kΩ∙µm were achieved at *V*_bg_ = 3 V. The cut-off frequency and maximum oscillation frequency can be improved by back-gate modulation. Extrinsic and intrinsic cutoff frequency of 6 and 19 GHz were demonstrated for a gate length of 190 nm at *V*_bg_ = 3 V. The intrinsic maximum oscillation frequency can become 2.1 times as high as the *f*_max_ without a back-gate bias. The results presented here indicate that tuning the back-gate voltage provides an effective way to boost *f*_T_ and *f*_max_ and give an insight into the high-frequency performance of MoS_2_ RF transistors.

## Figures and Tables

**Table 1:** Comparison of reported MoS_2_ RF transistors with comparable gate length.

MoS_2_	Substrate	*L*_g_ (nm)	*f*_T,intrinsic_	*f*_max,intrinsic_	References
			(GHz)	(GHz)	
Exfoliated	SiO_2_/Si	240	6	8.2	[22]
CVD	SiO_2_/Si	250	6.7	5.3	[23]
CVD	SiO_2_/Si	150	20	11.4	[24]
CVD	HfLaO/Si	190	19	29.7	This Work

## Data Availability

The data that support the findings of this study are available from the corresponding author upon reasonable request.
